# Ammonia Vapor-Induced Pseudomorphic Transformation of Mesoporous TiO_2_ Sol–Gel Coatings

**DOI:** 10.1021/acsomega.5c04483

**Published:** 2025-07-28

**Authors:** Adrienn Márta Bors, János Madarász, Norbert Nagy, Adél Sarolta Rácz, György Sáfrán, Dániel Olasz, Zoltán Hórvölgyi, Emőke Albert

**Affiliations:** † Department of Physical Chemistry and Materials Science, Budapest University of Technology and Economics, Műegyetem rkp. 3, Budapest 1111, Hungary; ‡ Department of Inorganic and Analytical Chemistry, Budapest University of Technology and Economics, Műegyetem rkp. 3, Budapest 1111, Hungary; § 303347 HUN-REN Centre for Energy Research, Institute of Technical Physics and Materials Science, Konkoly-Thege Miklós út 29-33, Budapest 1121, Hungary

## Abstract

This study reports the structural rearrangement of TiO_2_ sol–gel coatings via aqueous ammonia vapor-induced pseudomorphic transformation. The coatings were applied to glass, silica-coated glass, and silica-coated silicon substrates. The transformation was initiated by aging the freshly deposited coatings—still containing the molecular template Pluronic P123—in an aqueous ammonia vapor atmosphere, resulting in significant reorganization of the primarily formed structure. Compared to aqueous vapor treatment for 4 days, the ammonia-based approach was more effective in enhancing optical transmittance, yielding a 1.25% higher average increase after just 4 h. The transformation led to notable changes in material properties: the monolayer coatings exhibited increased open porosity (from 38% to 55%), higher thickness (from 130 to 205 nm), and a reduced specific surface area (from 713 m^2^/cm^3^ to 392 m^2^/cm^3^). Additionally, a slight increase in pore radius (from 5.6 to 6.6 nm) was observed. While the photocatalytic activity decreased under both UV and visible light due to reduced surface area, the improved optical performance highlights the potential of aqueous ammonia vapor treatment as a powerful tool for tailoring the structure and functionality of mesoporous TiO_2_ coatings.

## Graphical abstract

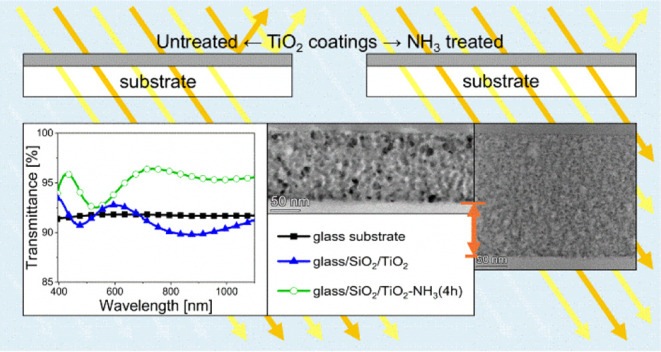

## Introduction

Mesoporous TiO_2_ coatings are widely employed in photovoltaic,^1–5^ photocatalytic,^6–12^ and optical^8^ applications due to their exceptional electronic and optical properties, chemical inertness, and remarkable photo- and thermal stability. Their cost-effectiveness further enhances their utility, rendering TiO_2_ indispensable in a multitude of advanced technological domains, including sensor technology,^13^ environmental remediation processes (such as air and water purification),^14^ and antimicrobial^15–17^ applications. The enhancement of light transmission has also attracted considerable interest, driven by its significance in optics, solar energy harvesting, and display technologies.^18^ Introducing mesopores into TiO_2_ coatings enables a reduction in the effective refractive index, thereby minimizing interfacial reflections and significantly improving light transmission.^19,20^ However, to achieve this, precise control of the porosity^21^ is required to ensure structural stability and homogeneity, while also achieving the targeted optical performance.

The pseudomorphic transformation of mesoporous sol–gel coatings in an aqueous vapor atmosphere provides a promising strategy for reducing their refractive index, increasing the size of the pores formed in the initial step, while preserving their mesoporous structure and optical transparency. Pseudomorphic transformation in sol–gel coatings refers to a solid-state dissolution–reprecipitation mechanism associated with Ostwald ripening, where the internal structure or phase composition changes while the external morphology is preserved.^22–24^ This transformation is used to improve the structural, optical, or catalytic properties of coatings without compromising their physical integrity and can be finely controlled by tuning the treatment conditions. In SiO_2_-based systems, pseudomorphic transformation can occur through treatments often involving ammonia and water vapor, leading to a reorganization of the internal pore structure.^22,23,25^

In the case of TiO_2_ sol–gel coatings, the number of studies investigating structural transformations under vapor-phase conditions remains remarkably limited. Existing reports mainly address pseudomorphic transformations occurring in a water vapor atmosphere, typically requiring prolonged exposure times, often ranging from 24 h to multiple days.^26–30^ Although the catalytic role of ammonia in the formation of titania (via hydrolysis and polycondensation) is well known,^31,32^ the pseudomorphic transformation of titania sol–gel coatings in the presence of ammonia has not yet been investigated.

In this study, we explored the impact of aqueous ammonia vapor treatment on the structural rearrangement of mesoporous TiO_2_ sol–gel coatings. The mesoporous TiO_2_ coatings were deposited onto bare glass, compact silica-coated glass, or compact silica-coated silicon substrates by using the dip-coating technique. Colloidal aging, which supported the secondary structural evolution of the as-deposited layers, was carried out in an aqueous ammonia vapor atmosphere while the structure-directing surfactant molecules (Pluronic P123) were still present in the coating matrix. Structural rearrangement was studied and confirmed using various characterization techniques, including high-resolution transmission electron microscopy, UV-vis spectroscopy, and ellipsometric porosimetry, as well as investigations of Rhodamine 6G dye and silver uptake, along with photocatalytic activity measurements under both UV and visible light irradiation.

## Experimental Section

### Materials

The following materials were used in the experiments: titanium­(IV) isopropoxide (98+%, Acros Organics), tetraethyl orthosilicate (TEOS, 99%, Merck), hydrochloric acid (HCl, 37%, Reanal), ethanol (EtOH, g.r., > 99.8%, Lach-Ner), 2,4-pentanedione (99+%, Acros Organics), Pluronic P123 (P123, average mol wt 5800, 99%, Sigma-Aldrich), ammonium hydroxide (NH_4_OH, 25%, Lach-Ner), AgNO_3_ (99.8%, Lach-Ner), Rhodamine 6G (R6G, 95%, Sigma-Aldrich), and ultrapure water (18.2 MΩ·cm, purified with an Adrona Integrity+ filtration system).

Microscope glass slides (76 × 26 × 1 mm, Thermo Scientific, Menzel-Gläser) and silicon wafers (Siegert, (100), p-type, prime grade) were used as solid substrates for the coatings. Substrates were cleaned using 2-propanol (2-PrOH, for analysis, Carlo Erba) and ultrapure water. Prior to layer deposition, the cleaned silicon substrates underwent a 10-min O_2_ plasma treatment (radiofrequency-generated low-pressure plasma/SmartPlasma 10, Plasma Technology GmbH, coupled with an oxygen concentrator/DeVilbiss Healthcare 525 KS) to improve their adhesion and wettability with the ethanolic precursor sol.

2-Propanol (a.r. > 99.7%, Reanal, Hungary) was used as the adsorptive material during the ellipsometric porosimetry measurements.

All materials were of analytical grade and were used without further purification.

### Synthesis of Precursor Sols

Mesoporous titania coatings were prepared from an ethanolic precursor sol containing a Pluronic P123 template. After the complete dissolution of P123 in ethanol, titanium­(IV) isopropoxide and 2,4-pentanedione were added to the solution. After stirring at 25 °C for 1 h, ultrapure water was added, followed by 30 min of ultrasound treatment and a further 1 h of stirring at 25 °C. The molar ratios for titanium­(IV) isopropoxide: EtOH:2,4-pentanedione:H_2_O:P123 were 1:33.95:0.97:2.21:0.034.^33^

Compact silica barrier coatings were prepared from the precursor sol synthesized by acid-catalyzed, controlled hydrolysis and polycondensation of tetraethyl orthosilicate in ethanolic media. 0.1 M aqueous solution of hydrochloric acid was used as the catalyst. The molar ratios for TEOS:EtOH:H_2_O:HCl were 1:4.68:3.96:7 × 10^–3^, and the mixture was stirred at 25 °C for 1 h.^34^

### Preparation of the Samples

Sol–gel coatings on solid substrates (glass, silica-coated glass, and silica-coated silicon) were prepared from the precursor sols by the dip-coating method.

For a subset of the samples, a compact SiO_2_ barrier layer was initially deposited onto the glass substrates to inhibit the diffusion of Na^+^ ions into the TiO_2_ coatings, as Na^+^ ion diffusion during heat treatment is known to reduce the photocatalytic activity of TiO_2_.^35^ In addition, the presence of Na^+^ ions within the coatings may also influence the ion and molecule adsorption capacities of the pore system.

Cleaned and dried substrates were immersed in the precursor sols and pulled out at a constant speed of 6 cm/min (SiO_2_ precursor sol) or 12 cm/min (TiO_2_ precursor sol) by a dip-coater device (Plósz Mérnökiroda Kft., Hungary). Samples were heat-treated in a drying oven (Nabertherm B170) at 450 °C for 30 min (SiO_2_), with a heating rate of 21 °C/min, or at 480 °C for 1 h (TiO_2_), with a heating rate of 5 °C/min.

The colloidal aging of the TiO_2_ coatings in an aqueous ammonia vapor atmosphere was performed as follows: immediately after layer deposition, a subset of the P123-containing TiO_2_ samples was placed in a sealed desiccator above a 2 M aqueous ammonium hydroxide solution at ambient temperature (22–26 °C) and exposed to aqueous ammonia vapor for 1, 2, 3, 4, or 6 h.^24,36^ The volume of the desiccator was 11 L. Afterward, the samples were heat-treated at 480 °C for 1 h with a heating rate of 5 °C/min.

To provide a basis for the future assessment of antibacterial activity, as well as to obtain indirect evidence of the surface and structure modification resulting from ammonia vapor treatment, silver-modified TiO_2_ coatings were also prepared by impregnating the porous network of the TiO_2_ coatings with 0.03 M aqueous AgNO_3_ solution. This concentration was chosen based on our previous results.^16^ The impregnation was carried out with a dip-coater, applying a dipping speed of 1 cm/min, followed by withdrawing the samples at 12 cm/min after 2 min. The samples were then rinsed with distilled water, dried, and heat-treated at 60 °C for 30 min, followed by heating at 450 °C for 30 min, with a heating rate of 5 °C/min. The heat treatment was carried out to reduce the silver ions to metallic silver.^16^

In order to obtain indirect information regarding the structural rearrangement and to perform photocatalytic studies, Rhodamine 6G dye molecules were adsorbed into the pore system of the TiO_2_ coatings. The dyes were adsorbed from 2.5 × 10^–3^ M aqueous solutions at pH 10 using a dip-coater, at 25 °C. To promote the adsorption of dye molecules, the pH was adjusted with NH_4_OH. Samples were immersed and withdrawn 2 min later, at a rate of 10 cm/min. Finally, they were rinsed with ultrapure water and dried at room temperature.^7^

Powder samples were prepared for modeling the TiO_2_ coatings: precursor sols were dried for 2 h until the evaporation of the dispersion medium. Then, one of the samples was placed in a desiccator above an aqueous ammonium hydroxide solution (2 M) at room temperature (22–26 °C) for 4 h. Finally, both samples were heat-treated at 480 °C for 1 h.

### Investigation Methods

#### X-Ray Diffraction (XRD)

Crystallinity of the TiO_2_ powder samples was characterized by an X-ray diffractometer (Philips PANalytical X’pert Pro, with Cu Kα radiation) and X’celerator detector. Measurements were carried out in the 2Θ = 4°–84° range, with a scanning rate of 20 s/step and a step size of 0.0167°, applying the automatic divergence slit system with an irradiated length of 15 mm.

#### X-Ray Photoelectron Spectroscopy (XPS)

The surface chemical composition of the samples at a depth of 5–10 nm was measured by XPS on a ThermoFisher Scientific Escalab Xi^+^ instrument. The spectra were recorded using an Al Kα X-ray source (1486.6 eV), with an X-ray spot size of 900 μm, and a charge neutralizer was applied. Since the titanium peak is highly sensitive to monatomic argon sputtering,^37^ the pristine samples were gently cleaned after initial measurement using an argon gas cluster source with 4 keV energy and a cluster size of 1000. Survey spectra were measured in 0.5 eV steps with a 10 ms dwell time per data point. The following high-resolution spectra (in decreasing binding energies) were measured: silver Auger (MN1), sodium (1s), oxygen (1s), sodium Auger (KL1), titanium (2p), silver (3d), and carbon (1s) at 20 eV pass energies (providing a resolution of 0.6 eV) with 0.1 eV steps and a 50 ms dwell time per data point. Data evaluation was performed with ThermoAvantage software. Atomic concentrations were calculated from the peak areas after background removal and applying the sensitivity factor library (Althermo). Due to the use of an aluminum anode as the excitation source, the Na 1s peak overlapped with titanium Auger peaks; therefore, the sodium content was determined from the Auger peak. The carbon content was excluded from the calculation as it was present only as a surface contamination layer, which is usual for samples exposed to air.

#### UV–Visible Spectroscopy (UV–Vis)

Optical properties of coatings on glass substrates were measured using UV–vis spectroscopy. Transmittance spectra of the bare glass substrates and the coated samples were obtained using an Analytik Jena Specord 200–0318 UV–vis spectrophotometer in the wavelength range of 350–1100 nm, with a resolution of 1 nm and a scanning speed of 10 nm/s. The obtained transmittance curves were analyzed using thin-layer optical models. Transmittance spectra of the coatings were fitted with a homogeneous layer model, assuming a perpendicular angle of incidence and identical homogeneous monolayers of the same material on both sides of the transparent substrate (Hild model).^38^ For the SiO_2_/TiO_2_ type two-layered samples, a two-layered optical model was applied. The fitting procedures provided effective refractive index values (at 632.8 nm) and layer thickness values. The fitting procedure used a Levenberg–Marquardt algorithm. The porosity of the TiO_2_ coatings, based on the obtained effective refractive index values, was estimated using the Lorentz–Lorenz formula, considering a refractive index value of 2.400 for bulk TiO_2_.^39^

The light transmission increase of the samples, compared to that of their bare glass substrates, was characterized by the average transmittance increase (ATI). ATI was calculated from the difference in the integrated area of the transmittance spectra measured on the coated sample and the substrate in the wavelength range of 400–800 nm^36^ as presented by eq 1.

1 $$\mathrm{ATI}=\frac{\overset{800}{\underset{i=400}{\sum }}\left(T_{\mathrm{sample}}-T_{\mathrm{substrate}}\right)}{800-400}$$

#### Ellipsometric Porosimetry (EP)

Ellipsometric porosimetry was used to determine the open porosity, pore size, and pore size distribution of the aqueous ammonia vapor-treated and (for comparison) the nontreated TiO_2_ coatings deposited directly on glass or silica-coated glass substrates. The measurements were carried out using Semilab’s Sopra EP-12 ellipsometric porosimeter. EP is a technique suitable for characterizing porous thin coatings in terms of open porosity, surface area, pore size, and mechanical strength. EP is a coupled technique in which vapor adsorption can be studied step-by-step through spectroscopic ellipsometry. During the measurement, vapor of the adsorptive material condenses in the pore system, which induces an effective refractive index shift. By measuring the adsorption and desorption isotherms, the porosity of the sample can be determined; furthermore, the pore size distribution can be calculated based on the modified Kelvin equation in the case of mesopores. The TiO_2_ coatings were characterized by this method using 2-PrOH as the adsorptive material. Before the measurement, the samples were heated to 175 °C for 15 min, and then vacuumed. The 2-propanol adsorption–desorption isotherms were measured at 24 °C. EP also provides the possibility to determine the optical properties of the samples as a spectroscopic ellipsometer at zero relative pressure of the adsorptive material. The measurements were carried out at an angle of incidence of 60° in the wavelength range of 248.5–971.5 nm. The thickness and the effective refractive index (at 632.8 nm) of the coatings were determined by applying the Tauc–Lorentz oscillator model. The specific surface area values were determined using the BET method, taking into account a molecular cross-sectional area of 0.388 nm^2^ for 2-PrOH.^40^

#### Transmission Electron Microscopy (TEM)

The structures of the aqueous ammonia vapor-treated and untreated TiO_2_ coatings, undoped or doped with silver, deposited on silica-coated silicon substrates, were investigated by TEM using a Thermo Fisher (Waltham, MA, USA) Titan Themis 200 kV spherical aberration (Cs)-corrected TEM/STEM microscope, which has a 0.08 nm high-resolution TEM and a 0.16 nm scanning TEM point resolution, and is equipped with 4 Super-X EDS detectors. Samples on silicon substrates were prepared in cross-section for TEM investigation by conventional mechanical and ion beam thinning techniques.

### Photocatalytic Investigations

For getting indirect information about the structural changes, photocatalytic experiments were carried out at the air–solid interface. R6G dye-impregnated coatings were irradiated with UV and visible light. Two 20 W UV-A light bulbs (Sylvania BL368, light emission maximum at 368 nm) were used as the UV radiation source, and two 57 W LED light bulbs (GE 93845, spectrum: emitting light from 420 to 780 nm with a maximum at 600 nm) were used as the visible light source. The two light bulbs and the measured samples were placed in an irradiation house. The samples were positioned halfway between the two light sources, with the light arriving at the coating perpendicularly from both sides, at an incident light intensity of 0.20 mW/cm^2^ and 0.47 mW/cm^2^ in the UV and visible light sources, respectively. The distances between the sample and the light sources were 18 cm (for UV lamps) and 24 cm (for visible light-emitting lamps). Dye photodegradation was monitored by measuring the absorbance of the dye-impregnated coatings after a period of UV or visible irradiation in the 400–800 nm wavelength range. The absorption spectra were measured by an Analytik Jena Specord 200-0318 type UV–vis spectrophotometer. The absorption spectra were corrected with the absorbance values measured before dye adsorption. The absorbance change of different samples was compared by calculating *A*/*A*_0_ where *A* and *A*_0_ are the absorbance values at around 533 nm (measured at the maximum of the absorbance spectra, corresponding to the R6G dye monomer peak)^7,41,42^ after *t* time of irradiation and before irradiation, respectively.

## Results and Discussion

### Characterization of the Samples by XRD

Untreated and ammonia vapor-treated TiO_2_ powder samples were prepared from the precursor sol used for layer deposition, and their XRD patterns are presented in Figure 1. As expected based on the applied annealing temperature during sample preparation, all XRD peaks are attributed to the anatase [PDF 98-000-9852] crystalline phase of TiO_2_ for both samples (Figure 1, black and red patterns). It is noteworthy that the aqueous ammonia vapor-treated sample exhibited reduced intensity, which suggests a decreased degree of crystallinity. Average crystallite sizes were determined from the highest intensity peak (2θ = 25.57°, corresponding to the anatase (101) plane) of the XRD patterns using Scherrer’s equation. Practically the same crystallite sizes were obtained for the two samples: the average crystallite size was 9.96 nm and 9.91 nm for the untreated and ammonia vapor-treated TiO_2_, respectively.

**Figure 1:**
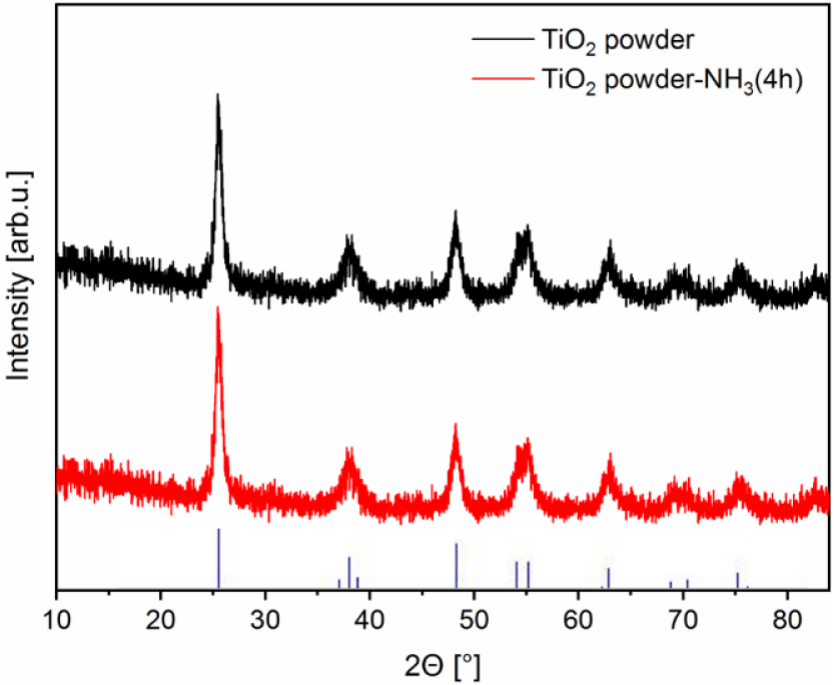
XRD patterns of the untreated TiO_2_ powder sample (black pattern) and the TiO_2_ powder sample treated under a 2 M aqueous NH_3_ solution atmosphere for 4 h (red pattern) are shown. The positions of the standard reflections for anatase [PDF 98-000-9852] are also shown as vertical bars at the bottom. The diagram is shifted along the intensity axis for better visibility.

### Characterization of the Samples by XPS

The surface chemical composition of the mono- and two-layered samples on glass substrates at a depth of 5–10 nm has been measured by XPS. The chemical composition values of the investigated samples are summarized in Table 1. To reveal the bonding states, the spectra were fitted by Gaussian–Lorentzian functions with a Gaussian contribution of 30%. The splitting value (energy difference between the 2p 1/2 and 2p 3/2 peaks) for titanium was found to be 5.7 eV, which corresponds to TiO_2_.^43^ The charging was corrected by setting the Ti 2p 3/2 value to 458.5 eV (Figure SI1).

**Table 1:** Chemical Composition of TiO_2_ Samples in 5–10 nm Depth before and after Cluster Sputtering

Sample (before/after cluster sputtering)	Ti [at. %]	O [at. %]	Na [at. %]	Ag [at. %]
glass/TiO_2_ (before cluster sputtering)	27.50	62.20	10.30	-
glass/TiO_2_ (after cluster sputtering)	28.30	61.50	10.20	-
glass/TiO_2_-Ag(0.03M) (before cluster sputtering)	27.40	61.80	10.70	0.06
glass/TiO_2_-Ag(0.03M) (after cluster sputtering)	28.80	61.70	9.40	0.07
glass/TiO_2_-NH_3_(4h) (before cluster sputtering)	26.50	61.30	12.20	-
glass/TiO_2_-NH_3_(4h) (after cluster sputtering)	27.70	61.60	10.70	-
glass/TiO_2_-NH_3_(4h)-Ag(0.03M) (before cluster sputtering)	27.90	61.70	10.30	0.09
glass/TiO_2_-NH_3_(4h)-Ag(0.03M) (after cluster sputtering)	28.50	61.60	9.80	0.09
glass/SiO_2_/TiO_2_ (before cluster sputtering)	30.70	66.70	2.60	-
glass/SiO_2_/TiO_2_ (after cluster sputtering)	32.50	65.90	1.60	-
glass/SiO_2_/TiO_2_-Ag(0.03M) (before cluster sputtering)	31.10	65.80	3.10	0.07
glass/SiO_2_/TiO_2_-Ag(0.03M) (after cluster sputtering)	32.50	65.80	1.60	0.07
glass/SiO_2_/TiO_2_-NH_3_(4h) (before cluster sputtering)	30.50	66.20	3.30	-
glass/SiO_2_/TiO_2_-NH_3_(4h) (after cluster sputtering)	32.10	65.80	2.10	-
glass/SiO_2_/TiO_2_-NH_3_(4h)-Ag(0.03M) (before cluster sputtering)	30.70	67.10	1.90	0.30
glass/SiO_2_/TiO_2_-NH_3_(4h)-Ag(0.03M) (after cluster sputtering)	31.80	66.30	1.60	0.30

Although the compact SiO_2_ barrier layer did not entirely prevent Na^+^ ion diffusion, it significantly reduced their concentration within the TiO_2_ coating: the monolayered TiO_2_ coatings, deposited directly on the glass substrates, contain a higher amount (9.4–12.2 at. %) of Na^+^ ions than those deposited on the surface of the compact SiO_2_ barrier layer (1.6–3.3 at. %) (Table 1).

In the case of monolayered samples, the silver content is slightly higher in the ammonia-treated sample (glass/TiO_2_-NH_3_(4h)-Ag­(0.03M)) compared to the untreated one (glass/TiO_2_-Ag­(0.03M)). No significant difference in silver content can be observed between the untreated monolayered (glass/TiO_2_-Ag­(0.03M)) and two-layered (glass/SiO_2_/TiO_2_-Ag­(0.03M)) samples. However, a considerable difference can be observed in the case of two-layered samples: the glass/SiO_2_/TiO_2_-NH_3_(4h)-Ag­(0.03M) sample contains more than three times the amount of silver compared to the glass/SiO_2_/TiO_2_-Ag­(0.03M) sample. This may be attributed to the synergistic effect of the compact SiO_2_ barrier layer and the aqueous ammonia vapor treatment: the former contributes to the regulation of surface charge density within the pores, while the latter plays a crucial role in the development of pore structure and porosity.

The oxides of silver show a negative binding energy shift compared to the metal. The silver peaks could be fitted by 2 components: the component of higher binding energy, 368.3 ± 0.1 eV, belongs to metallic silver, while the lower binding energy component, 0.8 eV ± 0.1 eV energy difference, could be ascribed to oxidized silver species.^43–45^ An example of the peak decomposition for a glass/SiO_2_/TiO_2_-NH_3_(4h)-Ag­(0.03M) sample after cluster sputtering is shown in Figure SI2. Metallic silver is considered beneficial for photocatalytic enhancement,^46,47^ whereas both metallic and ionic silver contribute to antimicrobial activity.^48^ The relative contents of the metallic and ionic silver species for the silver-doped samples, along with their corresponding values expressed in atomic percent, are presented in Table 2. In all samples, silver is present in a higher proportion in ionic form than in its metallic form. 40% of the silver is in metallic form in the glass/TiO_2_-Ag­(0.03M) and glass/SiO_2_/TiO_2_-NH_3_(4h)-Ag­(0.03M) samples, whereas only 10% and 30% is metallic in the glass/TiO_2_-NH_3_(4h)-Ag­(0.03M) and glass/SiO_2_/TiO_2_-Ag­(0.03M) samples, respectively (Table 2). The highest atomic percentage of metallic silver was observed in the sample glass/SiO_2_/TiO_2_-NH_3_(4h)-Ag­(0.03M), while the lowest atomic percentage was detected in glass/TiO_2_-NH_3_(4h)-Ag­(0.03M). The samples glass/TiO_2_-Ag­(0.03M) and glass/SiO_2_/TiO_2_-Ag­(0.03M) exhibited comparable metallic silver contents.

**Table 2:** Relative Contents of Silver Species (Metallic and Ionic) after Cluster Sputtering and Their Corresponding Values Expressed in Atomic Percent

Sample	Metallic silver (rel. cont.) [-]	Ionic silver (rel. cont.) [-]	Metallic silver [at. %]	Ionic silver [at. %]
**glass/TiO**_**2**_ **-** **Ag(0.03M)**	0.4	0.6	0.028	0.042
**glass/TiO**_**2**_ **-** **NH**_**3**_ **(4h)** **-Ag(0.03M)**	0.1	0.9	0.009	0.081
**glass/SiO**_**2**_ **/** **TiO**_**2**_ **-Ag(0.03M)**	0.3	0.7	0.021	0.049
**glass/SiO**_**2**_ **/** **TiO**_**2**_ **-** **NH**_**3**_ **(4h)** **-Ag(0.03M)**	0.4	0.6	0.12	0.18

### Optical Properties of the Samples: UV–Vis Spectroscopy

The light transmittance of the ammonia-treated and (for comparison) untreated mono- and two-layered samples on glass substrates was investigated by UV–vis spectroscopy. Thickness and effective refractive index values of the undoped monolayered (glass/TiO_2_, glass/TiO_2_-NH_3_) and two-layered (glass/SiO_2_/TiO_2_, glass/SiO_2_/TiO_2_-NH_3_) sol–gel coatings were determined based on thin-layer optical models by applying fitting procedures (Tables 3 and 4).^39^ Porosity values, calculated by the Lorentz–Lorenz formula in view of the effective refractive index of the TiO_2_-coatings and the refractive index of bulk TiO_2_ (*n*_bulk_ = 2.400) and air (*n*_air_ = 1), are also presented in Tables 3 and 4.^39^

**Table 3:** Effective Refractive Index (*n*), Thickness (*d*), Calculated Porosity (*P*, Lorentz–Lorenz) Values of the TiO_2_ Coatings of the glass/SiO_2_/TiO_2_-NH_3_-Type Samples, Treated in Aqueous Ammonia Vapor Atmosphere for Different Durations (1, 2, 3, 4, and 6 h), Determined from Their UV-Vis Transmittance Spectra^a^

Sample	*n* [-] (632.8 nm) TiO_2_	*d* [nm] TiO_2_	P [%] TiO_2_	ATI [%] (400–800 nm)
**glass/SiO**_**2**_ **/** **TiO**_**2**_	1.5326 ± 0.0058	158 ± 2	49 ± 0	0.53 ± 0.10
**glass/SiO**_**2**_ **/** **TiO**_**2**_ **-** **NH**_**3**_ **(1h)**	1.4533 ± 0.0017	165 ± 9	56 ± 0	2.00 ± 0.08
**glass/SiO**_**2**_ **/** **TiO**_**2**_ **-** **NH**_**3**_ **(2h)**	1.4286 ± 0.0058	170 ± 2	58 ± 0	2.59 ± 0.14
**glass/SiO**_**2**_ **/** **TiO**_**2**_ **-** **NH**_**3**_ **(3h)**	1.4161 ± 0.0075	178 ± 7	59 ± 1	2.90 ± 0.03
**glass/SiO**_**2**_ **/** **TiO**_**2**_ **-** **NH**_**3**_ **(4h)**	1.3763 ± 0.0044	204 ± 3	63 ± 0	3.25 ± 0.06
**glass/SiO**_**2**_ **/** **TiO**_**2**_ **-** **NH**_**3**_ **(6h)**	1.3883 ± 0.0018	199 ± 2	62 ± 0	3.16 ± 0.03

a The table also contains the average transmittance increment (ATI) values of the two-layered samples , determined for the 400–800 nm wavelength range, compared to their bare glass substrates. For comparison, the results obtained for the untreated sample (glass/SiO_2_/TiO_2_) are also presented .

**Table 4:** Effective Refractive Index (*n*), Thickness (*d*), and Calculated Porosity (*P*, Lorentz–Lorenz) Values of the Investigated Samples, Determined from Their UV-Vis Transmittance Spectra

Sample	*n* [-] (632.8 nm) SiO_2_	*d* [nm] SiO_2_	*n* [-] (632.8 nm) TiO_2_	*d* [nm] TiO_2_	*P* [%] TiO_2_
**glass/TiO**_**2**_	-	-	1.5506 ± 0.0090	149 ± 5	48 ± 1
**glass/TiO**_**2**_ **-** **NH**_**3**_ **(4h)**	-	-	1.3407 ± 0.0041	248 ± 4	66 ± 0
**glass/SiO**_**2**_ **/** **TiO**_**2**_	1.4433 ± 0.0012	216 ± 6	1.5326 ± 0.0058	158 ± 2	49 ± 0
**glass/SiO**_**2**_ **/** **TiO**_**2**_ **-NH**_**3**_ **(4h)**	1.4282 ± 0.0015	193 ± 3	1.3763 ± 0.0044	204 ± 3	63 ± 0

The effect of the aqueous ammonia vapor treatment’s duration on the light transmittance of the two-layered glass/SiO_2_/TiO_2_-NH_3_-type samples is shown Figure 2a. It can be observed that all of the representative transmittance curves indicate an increase in light transmission compared to the bare glass substrate. Additionally, the light transmittance increases with the duration of ammonia treatment from 1 to 4 h, followed by a slight decrease (compare the transmittance spectra of glass/SiO_2_/TiO_2_-NH_3_(4h) and glass/SiO_2_/TiO_2_-NH_3_(6h) samples). This trend suggests that the pseudomorphic transformation induced by the aqueous ammonia vapor atmosphere facilitates enhanced light transmission, with an optimum in treatment duration. The increase in layer thickness, the reduction in effective refractive index, and the associated increase in porosity as a function of aqueous ammonia vapor treatment duration (ranging from 1 to 4 h) are in strong agreement with the trends observed in the transmittance spectra (Figure 2a and Table 3). Based on these results, a treatment duration of 4 h was selected as the optimal condition for subsequent ammonia vapor exposure experiments.

**Figure 2:**
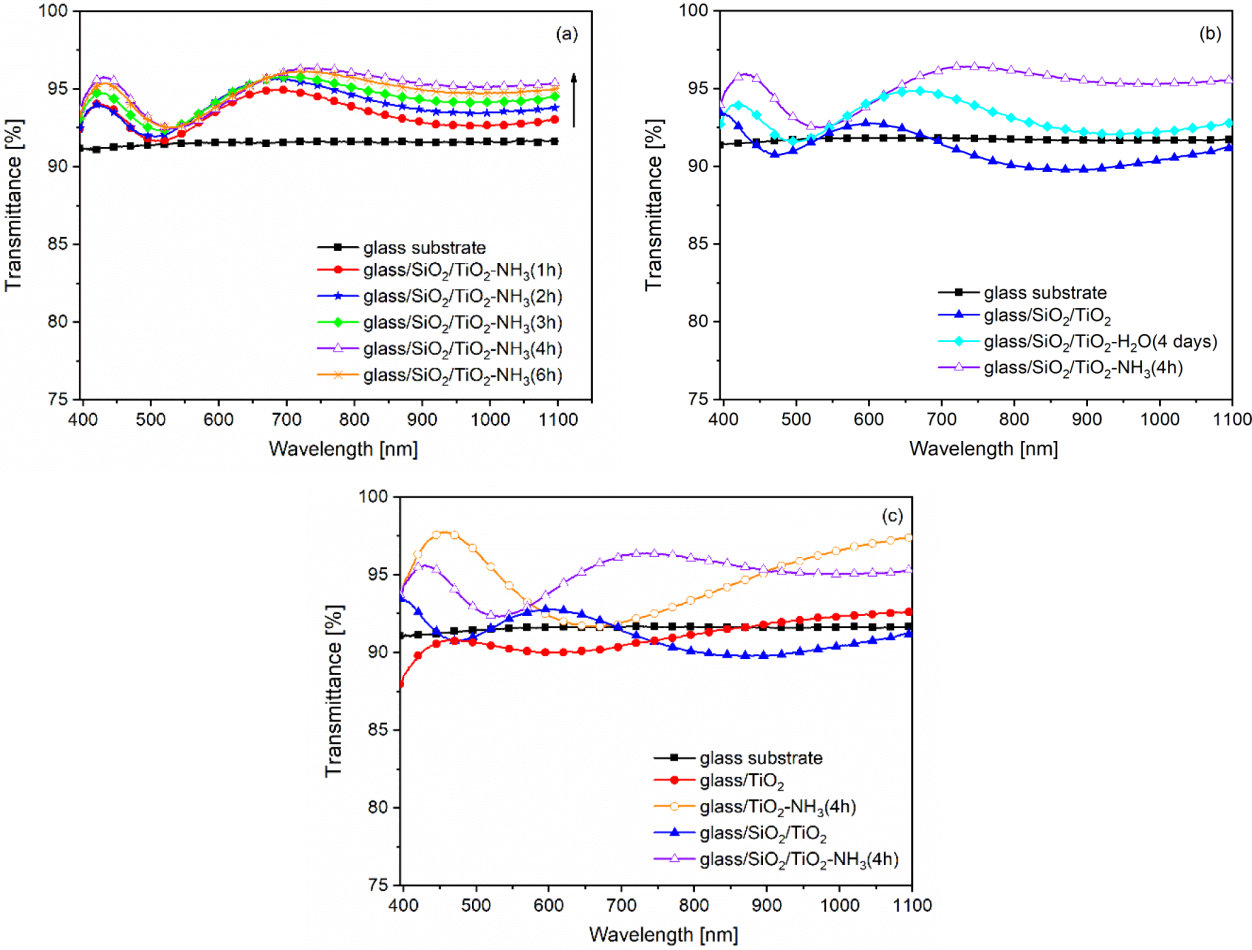
Representative transmittance spectra of the bare glass substrate and the two-layered SiO_2_/TiO_2_-NH_3_-type samples treated in an aqueous ammonia vapor atmosphere for different durations (1, 2, 3, 4, and 6 h) (a), the two-layered SiO_2_/TiO_2_, SiO_2_/TiO_2_-H_2_O­(4 days), and SiO_2_/TiO_2_-NH_3_(4h) samples (b), and the monolayered (glass/TiO_2_, glass/TiO_2_-NH_3_(4h)) and two-layered (glass/SiO_2_/TiO_2_, glass/SiO_2_/TiO_2_-NH_3_(4h)) samples on glass substrates, untreated (glass/TiO_2_, glass/SiO_2_/TiO_2_) or treated (glass/TiO_2_-NH_3_(4h), glass/SiO_2_/TiO_2_-NH_3_(4h)) in an aqueous ammonia vapor atmosphere for 4 h after layer deposition (c).

For comparative purposes, the two-layered sample was also exposed to a saturated water vapor atmosphere for varying durations. Among the conditions tested, the treatment conducted over a period of 4 days resulted in the most pronounced enhancement in light transmittance compared with the bare glass substrate. Figure 2b compares the transmittance spectra of the untreated two-layered (glass/SiO_2_/TiO_2_) sample, the two-layered sample treated in an aqueous ammonia vapor atmosphere for 4 h (glass/SiO_2_/TiO_2_-NH_3_(4h)), and the two-layered sample treated in a water vapor atmosphere for 4 days (glass/SiO_2_/TiO_2_-H_2_O­(4 days)). It can be observed that the 4-h treatment in an aqueous ammonia vapor atmosphere led to a more pronounced increase in light transmittance (ATI = 3.25 ± 0.07%) than the 4-day aqueous vapor treatment (ATI = 2.00 ± 0.19%) (Table 5).

**Table 5:** Maximum Transmittance (*T*_max_) Values and Average Transmittance Increase (ATI) of the Investigated Samples in the 400–800 nm Wavelength Range, Compared to Their Bare Glass Substrates, and Their Transmittance at 550 Nm^a^

Sample	*T*_max_ [%] (400–800 nm)(λ*T*_max_)	*T* [%] (550 nm)	ATI [%] (400–800 nm)
**glass/SiO**_**2**_	95.3 ± 0.01 (400 nm)	92.1 ± 0.05	1.51 ± 0.05
**glass/TiO**_**2**_	91.8 ± 0.31 (800 nm)	90.7 ± 0.25	–0.87 ± 0.21
**glass/TiO**_**2**_ **-** **NH**_**3**_ **(4h)**	98.3 ± 0.11 (446–460 nm)	94.4 ± 0.37	2.40 ± 0.08
**glass/SiO**_**2**_ **/** **TiO**_**2**_	94.3 ± 0.16 (445–457 nm)	91.4 ± 0.12	0.53 ± 0.10
**glass/SiO**_**2**_ **/** **TiO**_**2**_ **-NH**_**3**_ **(4h)**	96.5 ± 0.10 (719–736 nm)	92.9 ± 0.10	3.25 ± 0.07
**glass/** **TiO**_**2**_ **-Ag(0.03M)**	91.1 ± 0.36 (800 nm)	89.7 ± 0.11	–1.56 ± 0.16
**glass/TiO**_**2**_ **-** **NH**_**3**_ **(4h)-Ag(0.03M)**	96.8 ± 0.10 (454–468 nm)	93.6 ± 0.36	1.82 ± 0.05
**glass/SiO**_**2**_ **/TiO**_**2**_ **-** **Ag(0.03M)**	93.7 ± 0.13 (445–457 nm)	91.2 ± 0.13	0.45 ± 0.14
**glass/SiO**_**2**_ **/TiO**_**2**_ **-** **NH**_**3**_ **(4h)** **-Ag(0.03M)**	96.1 ± 0.07 (730–740 nm)	92.2 ± 0.12	2.85 ± 0.10
**glass/SiO**_**2**_ **/TiO**_**2**_ **-** **H**_**2**_ **O(** **4 days)**	95.0 ± 0.31(650–680 nm)	92.8 ± 0.92	2.00 ± 0.19

a Glass/SiO_2_ refers to the barrier compact SiO_2_ layer between the glass substrate and the TiO_2_ coating.).

The effect of aqueous ammonia vapor treatment on the light transmittance of the mono- and two-layered samples can be seen in Figure 2c. The light transmittance increase resulting from the aqueous ammonia vapor treatment can be observed for both monolayered and two-layered samples. Aging of the samples in an ammonia vapor atmosphere led to a reduction in the effective refractive index, along with an increase in both porosity and layer thickness values (Table 4). The ATI values changed from −0.87 ± 0.21% to 2.40 ± 0.08% and from 0.53 ± 0.10% to 3.25 ± 0.07% for the monolayered and two-layered samples, respectively (Table 5).

For comparison, samples from TiO_2_ precursor sols without the Pluronic P123 template were also prepared. The optical properties of the mono- and two-layered coatings deposited onto glass substrates, treated and untreated in an aqueous ammonia vapor atmosphere for 4 h, were determined based on their UV–vis transmittance spectra. The transmittance spectra of the samples prepared from precursor sols, both with and without Pluronic P123, are compared in Figure SI3. Table SI1 presents the layer thickness, effective refractive index, and porosity values determined for the TiO_2_ coatings, as well as the average transmittance increment (ATI) values of the samples.

It can be observed that, in contrast to the samples prepared from precursor suspensions containing Pluronic P123, the light transmittance of all samples derived from precursor suspensions without Pluronic P123 is significantly lower than that of the bare glass substrate (Figure SI3). This is manifested in the negative values of the average transmittance increment for these samples (Table SI1). The underlying reason is that, in the absence of Pluronic P123, micelle formation does not occur within the coatings, resulting in significantly lower porosity (see the porosity values in Table SI1). Notably, the aqueous ammonia vapor treatment exerted no significant influence on either the light transmittance or the optical parameters of the samples prepared from precursor suspensions lacking Pluronic P123 (Table SI1). This finding is particularly important, as it further reinforces our hypothesis regarding structural rearrangement via aqueous ammonia vapor-induced pseudomorphic transformation in mesoporous TiO_2_ sol–gel coatings prepared from precursor sol containing the Pluronic P123 micelle-forming agent.

The effect of silver doping on the light transmittance of the porous TiO_2_ coatings is shown in Figure 3. It can be observed that silver incorporation does not significantly influence the light transmittance of the samples. ATI values were reduced by 0.69%, 0.58%, 0.08%, and 0.4% for glass/TiO_2_, glass/TiO_2_-NH_3_(4h), glass/SiO_2_/TiO_2_, and glass/SiO_2_/TiO_2_-NH_3_(4h) samples, respectively (see also Table 5).

**Figure 3:**
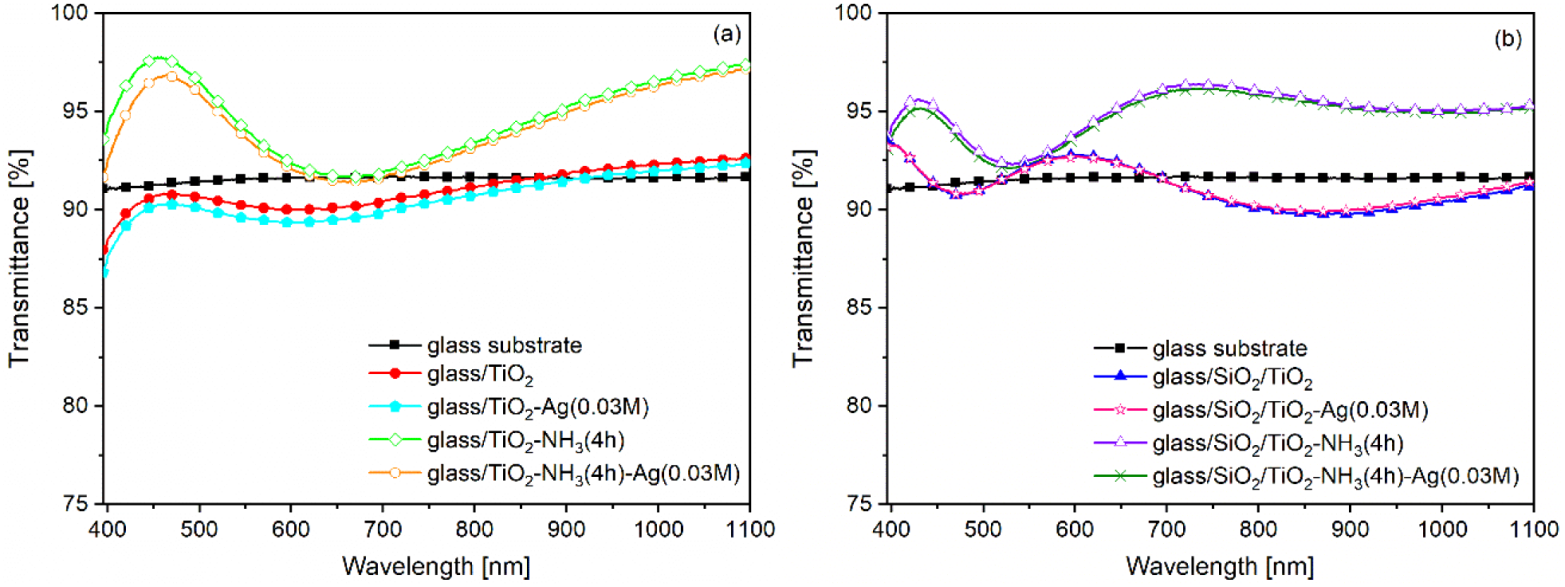
Transmittance spectra of the bare glass substrate and the aqueous ammonia vapor-treated (glass/TiO_2_-NH_3_(4h), glass/TiO_2_-NH_3_(4h)-Ag­(0.03M), glass/SiO_2_/TiO_2_-NH_3_(4h), glass/SiO_2_/TiO_2_-NH_3_(4h)-Ag­(0.03M)) and untreated (glass/TiO_2_, glass/TiO_2_-Ag­(0.03M), glass/SiO_2_/TiO_2_, glass/SiO_2_/TiO_2_-Ag­(0.03M)), monolayered (a), and two-layered (b) samples, not doped (glass/TiO_2_, glass/TiO_2_-NH_3_(4h), glass/SiO_2_/TiO_2_, glass/SiO_2_/TiO_2_-NH_3_(4h)) and doped (glass/TiO_2_-Ag­(0.03M), glass/TiO_2_-NH_3_(4h) -Ag­(0.03M), glass/SiO_2_/TiO_2_-Ag­(0.03M), glass/SiO_2_/TiO_2_-NH_3_(4h)-Ag­(0.03M)) with silver.

The highest efficiency in terms of enhanced light transmittance was exhibited by the glass/SiO_2_/TiO_2_-NH_3_(4h) two-layered sample. It is composed of a compact SiO_2_ bottom layer with a thickness of 193 ± 3 nm (refractive index of 1.4282 ± 0.0015) and a porous TiO_2_ top layer with a thickness of 204 ± 3 nm, 63% porosity (refractive index of 1.3763 ± 0.0044), maximum transmittance of 96.5 ± 0.10% (719–736 nm), and ATI value of 3.25 ± 0.07%. (see also Tables 4 and 5). It should be noted that, despite the higher *T*_max_ value of 98.3 ± 0.11% (446–460 nm) glass/TiO_2_-NH_3_(4h) type sample exhibited a significantly lower ATI (2.40 ± 0.08%) compared to the glass/SiO_2_/TiO_2_-NH_3_(4h) type sample. The transmittance values of the developed samples at 550 nm (a commonly referenced wavelength in the literature) are also presented in Table 5. To the best of our knowledge, no comparable results have been reported in the literature to date concerning TiO_2_ sol–gel coatings that exhibit transmittance values exceeding 90%—and in some cases approaching 94%—at a reference wavelength of 550 nm,^8^ nor regarding the structural rearrangement of the pore system of the TiO_2_ sol–gel coatings induced by ammonia vapor treatment. It is worth noting that the increase in light transmittance was significant even at the highest silver content (see the results for glass/SiO_2_/TiO_2_-NH_3_(4h)-Ag (0.03M) in Tables 2 and 5). This could further expand the potential for future applications (against pathogenic microorganisms).^16^

### Optical Properties of the Samples: Ellipsometric Porosimetry

The evolution of both the quantity and nature of the accessible open pores as a result of ammonia vapor treatment was investigated using ellipsometric porosimetry. The adsorption–desorption isotherms of the monolayered and two-layered samples deposited on glass substrates—both treated and, for comparison, untreated in an aqueous ammonia vapor atmosphere—are shown in Figure 4a,c,e,g. The measurements were conducted at 24 °C by using 2-propanol as the adsorbate. The normalized pore radius distributions of the pore systems presented in Figure 4b,d,f,h were determined by the modified Kelvin equation, assuming cylindrical pores. The obtained isotherms exhibit a type IV shape, while the hysteresis loops correspond to type H2, based on the IUPAC classification. This indicates the presence of mesoporous materials with an interconnected pore network and nonuniform pore sizes. The effective refractive index (at 632.8 nm) and the thickness of the coatings were also determined at zero relative pressure by using spectroscopic ellipsometry, applying the Tauc–Lorentz oscillator model for data fitting (Table 6). The total porosity of the samples was also determined using the Lorentz–Lorenz equation based on the effective refractive index values obtained at zero relative pressure (Table 6).

**Figure 4:**
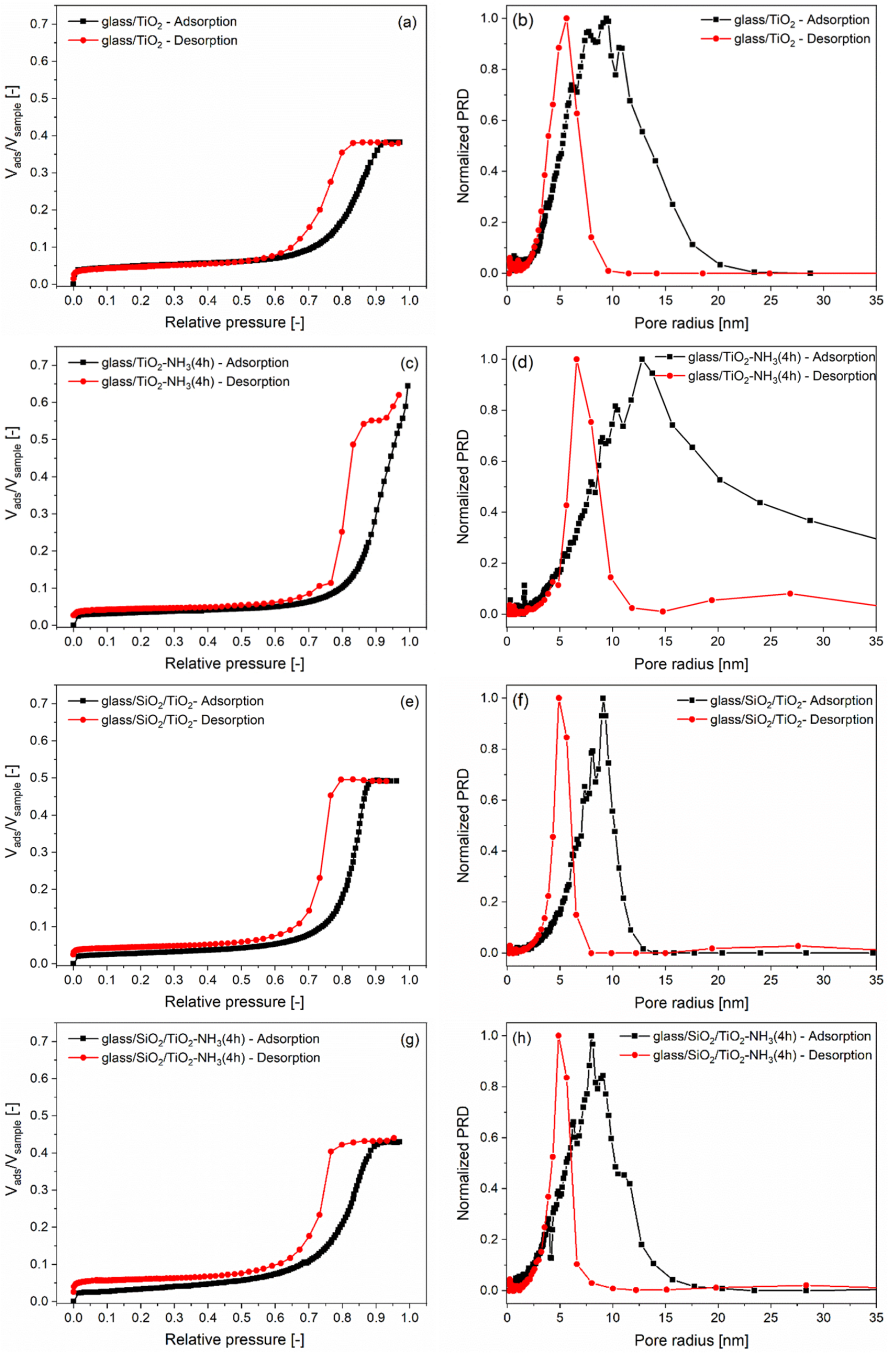
2-Propanol adsorption–desorption isotherms taken at 24 °C (a, c, e, g) and the corresponding normalized pore radius distributions (b, d, f, h) for the aqueous ammonia vapor-treated (c, d, g, h) and untreated (a, b, e, f) monolayered (a, b, c, d) and two-layered (e, f, g, h) samples on glass substrates, determined by ellipsometric porosimetry.

**Table 6:** Effective Refractive Index (*n*, 632.8 nm), Thickness (*d*), Total Porosity (Total *P*, Lorentz–Lorenz), and Open Porosity (Open *P*), Specific Surface Area (BET), and Pore Radius (*r*_ads_, *r*_des_) Values of Aqueous Ammonia Vapor (glass/TiO_2_-NH_3_(4h), glass/SiO_2_/TiO_2_-NH_3_(4h)) and Untreated (glass/TiO_2_, glass/SiO_2_/TiO_2_), Monolayered (glass/TiO_2_, glass/TiO_2_-NH_3_(4h)), and Two Layered (glass/SiO_2_/TiO_2_, glass/SiO_2_/TiO_2_-NH_3_(4h)) Samples on Glass Substrates, Determined by Ellipsometric Porosimetry

Sample	*n* [-] (632.8 nm) SiO_2_	*d* [nm] SiO_2_	*n* [-] (632.8 nm) TiO_2_	*d* [nm] TiO_2_	Total *P* [%]	Open P [%]	S_BET_ [m^2^/cm^3^]	r_ads_ [nm]	r_des_ [nm]
**glass/TiO**_**2**_	-	-	1.5920	130	45	38	713	9.4	5.6
**glass/TiO**_**2**_ **-** **NH**_**3**_ **(4h)**	-	-	1.3693	205	63	55	392	12.8	6.6
**glass/SiO**_**2**_ **/** **TiO**_**2**_	1.4368	203	1.5386	126	49	49	1156	9.1	4.9
**glass/SiO**_**2**_ **/** **TiO**_**2**_ **-** **NH**_**3**_ **(4h)**	1.3917	221	1.4367	190	57	43	966	7.9	4.9

The refractive index and total porosity values obtained by spectroscopic ellipsometry for the samples on glass substrates are in good agreement with those derived from the UV–vis transmittance spectra analysis (see Tables 4 and 6, 48–66% and 45–63% for total porosity, respectively).

Colloidal aging of the samples in the aqueous ammonia vapor atmosphere resulted in an increase in porosity (i.e., a decrease in the effective refractive index values) and a decrease in the specific surface area, along with an increase in the layer thickness value (Table 6). The enhancement in porosity resulting from the ammonia vapor atmosphere treatment correlates well with the pronounced increase in optical transmittance observed in the UV–vis spectra of the samples (Figure 2c and Table 4). The difference between total and open porosity values, as presented in Table 6, arises because some of the pores are either closed or inaccessible to the adsorptive molecules under the applied experimental conditions. During the ammonia vapor treatment, the fraction of open porosity relative to the total porosity remained nearly unchanged in the monolayered samples (glass/TiO_2_ vs glass/TiO_2_-NH_3_(4h)). In contrast, a notable decrease was observed in the two-layered samples (glass/SiO_2_/TiO_2_ vs glass/SiO_2_/TiO_2_-NH_3_(4h)) (Table 6). The ammonia vapor treatment resulted in a modest increase in pore size: the pore radius values determined from the desorption branch of the isotherm are 5.6 and 6.6 nm for the monolayered samples (glass/TiO_2_ and glass/TiO_2_-NH_3_(4h), respectively), and there was no change (4.9 nm) for the two-layered samples (glass/SiO_2_/TiO_2_ and glass/SiO_2_/TiO_2_-NH_3_(4h)).

### Characterization of the Samples by TEM

In Figure 5 the representative cross-sectional TEM images of the SiO_2_/TiO_2_ (a, b), SiO_2_/TiO_2_-Ag­(0.03M) (c, d, e), SiO_2_/TiO_2_-NH_3_(4h) (f, g), and SiO_2_/TiO_2_-NH_3_(4h)-Ag­(0.03M) (h, i, j) samples deposited on Si substrates are presented. Figure 5e,j were taken with the high-angle annular dark-field (HAADF) detector to visualize the silver particles and also display the STEM EDS elemental maps of the Ag particles.

**Figure 5:**
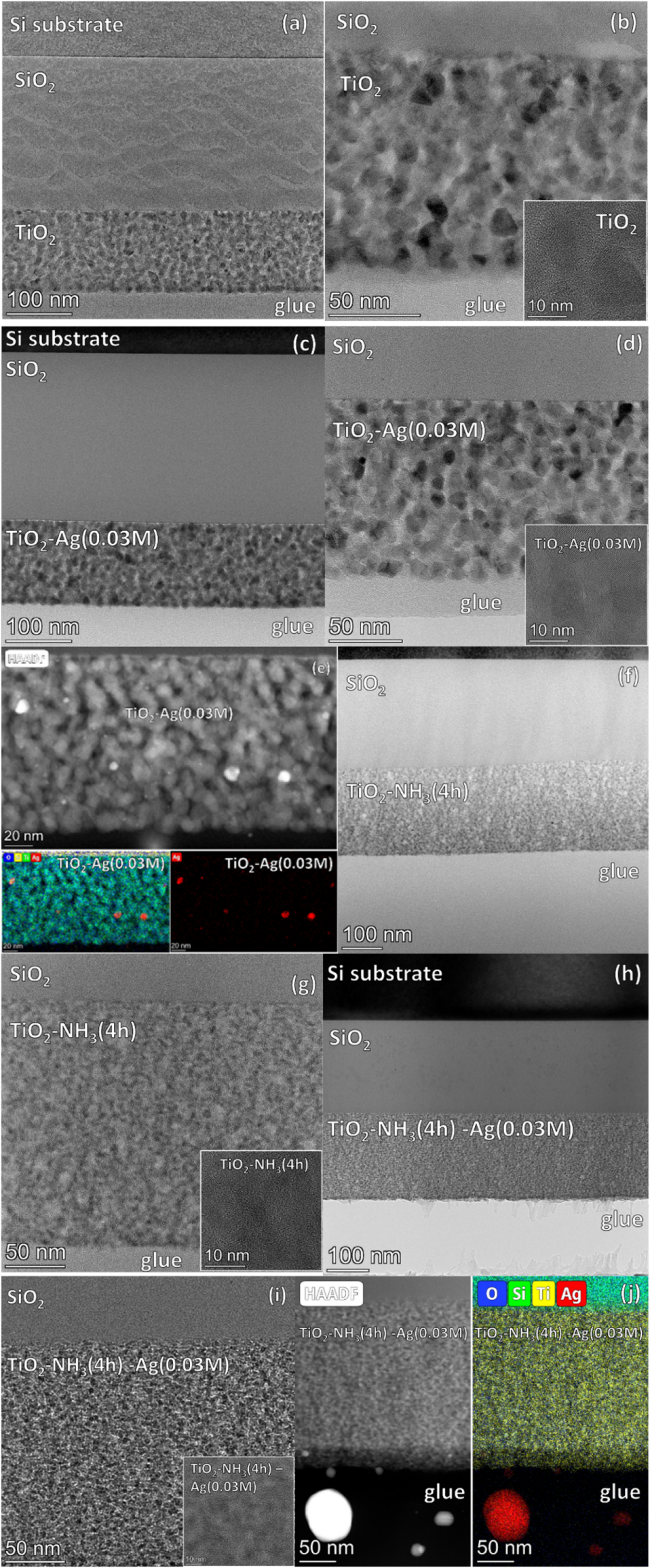
Cross-sectional TEM images of the SiO_2_/TiO_2_ (a, b), SiO_2_/TiO_2_-Ag­(0.03M) (c, d, e), SiO_2_/TiO_2_-NH_3_(4h) (f, g), and SiO_2_/TiO_2_-NH_3_(4h)-Ag­(0.03M) (h, i, j) type samples on Si substrates. Figures (e) and (j) were taken with the high-angle annular dark-field (HAADF) detector to visualize the silver particles and also display the STEM EDS elemental maps of Ag particles for the SiO2/TiO2-Ag­(0.03M) and SiO_2_/TiO_2_- NH3­(4h)-Ag­(0.03M) samples, respectively.

The TEM analysis, using both selected area electron diffraction and lattice-resolution HRTEM imaging, confirmed the presence of the anatase crystal phase in the investigated TiO_2_ samples, which is in line with the XRD results (Figure 1).

The layer thickness values (Table 7) are in good agreement with those obtained from optical characterization methods (UV–vis spectroscopy and ellipsometric porosimetry) for the same types of samples deposited onto glass substrates (see Tables 4, 6 and 7).

**Table 7:** Thickness Values of the Ammonia Vapor-Treated and Untreated, Silver-Doped and Undoped, Two-Layered Samples on Si Substrates, Determined from TEM Images

Sample	*d* [nm] SiO_2_	*d* [nm] TiO_2_
**Si/SiO**_**2**_ **/** **TiO**_**2**_	240 ± 2	120 ± 2
**Si/SiO**_**2**_ **/** **TiO**_**2**_ **-Ag(0.03M)**	261 ± 2	124 ± 1
**Si/SiO**_**2**_ **/TiO**_**2**_ **-** **NH**_**3**_ **(4h)**	271 ± 9	213 ± 2
**Si/SiO**_**2**_ **/** **TiO**_**2**_ **-** **NH**_**3**_ **(4h)** **-Ag(0.03M)**	226 ± 2	207 ± 2

The effect of structural rearrangement due to aqueous ammonia vapor treatment is evident in the TEM images: while the untreated SiO_2_/TiO_2_ (Figure 5a,b) and SiO_2_/TiO_2_-Ag (0.03M) (Figure 5c, d,e) samples exhibit a granular structure composed of TiO_2_ particles (typically 8–15 nm in size), the ammonia vapor-treated samples (SiO_2_/TiO_2_-NH_3_(4h) (Figure 5f,g), SiO_2_/TiO_2_-NH_3_(4h)-Ag (0.03M) (Figure 5h,i,j) display a much finer structure. Moreover, consistent with the findings from UV–vis spectroscopy and ellipsometric porosimetry, in addition to the structural rearrangement, an increase in coating thickness is also evident following the aqueous ammonia treatment (Table 7).

A significant difference was observed in the location, size, and distribution of the silver particles between the untreated (SiO_2_/TiO_2_-Ag (0.03M), Figure 5c,d,e) and ammonia vapor-treated (SiO_2_/TiO_2_-NH_3_(4h)-Ag (0.03M), Figure 5h,i,j) samples. The SiO_2_/TiO_2_-Ag (0.03M) coatings contain 5–10 nm Ag particles dispersed throughout the pore system and present at all depths (Figure 5c,d,e). In contrast, the ammonia vapor-treated sample (SiO_2_/TiO_2_-NH_3_(4h)-Ag (0.03M)) exhibits large, anisometric silver particles with a broad size distribution (13–70 nm) located on the surface, whereas no silver particles are present within the pore system (Figure 5h,i,j. This finding supports and explains the notable difference in surface silver content between the two samples, as revealed by XPS analysis (Table 1).

### Adsorption of Rhodamine 6G Dye and UV/Visible Light-Induced Photocatalytic Activity of Mesoporous TiO_2_ Samples

To obtain indirect information on the structural rearrangement induced by the ammonia vapor treatment of the samples, their dye adsorption capacity was investigated. The absorbance spectra of the R6G dye-impregnated samples are shown in Figure 6a, indicating structural differences among the various TiO_2_ coating types. Samples treated in an ammonia vapor atmosphere adsorbed 1.46 times (glass/TiO_2_-NH_3_(4h)) and 1.77 times (glass/SiO_2_/TiO_2_-NH_3_(4h)) more R6G dye molecules compared to the untreated samples. This enhancement is attributed to the increased thickness, porosity, and structural transformation of the mesoporous TiO_2_ coatings induced by the aqueous ammonia vapor treatment. Samples with a compact SiO_2_ barrier layer adsorbed a significantly lower amount of R6G dye molecules, presumably due to differences in surface charge density compared to coatings without the barrier layer, and to variations in sodium ion content, as evidenced by XPS analysis results (Table 1). The presence of silver slightly reduced the R6G dye uptake in the porous TiO_2_ coatings, likely due to the partial occupation of adsorption sites by silver particles (Figure 6a).

**Figure 6:**
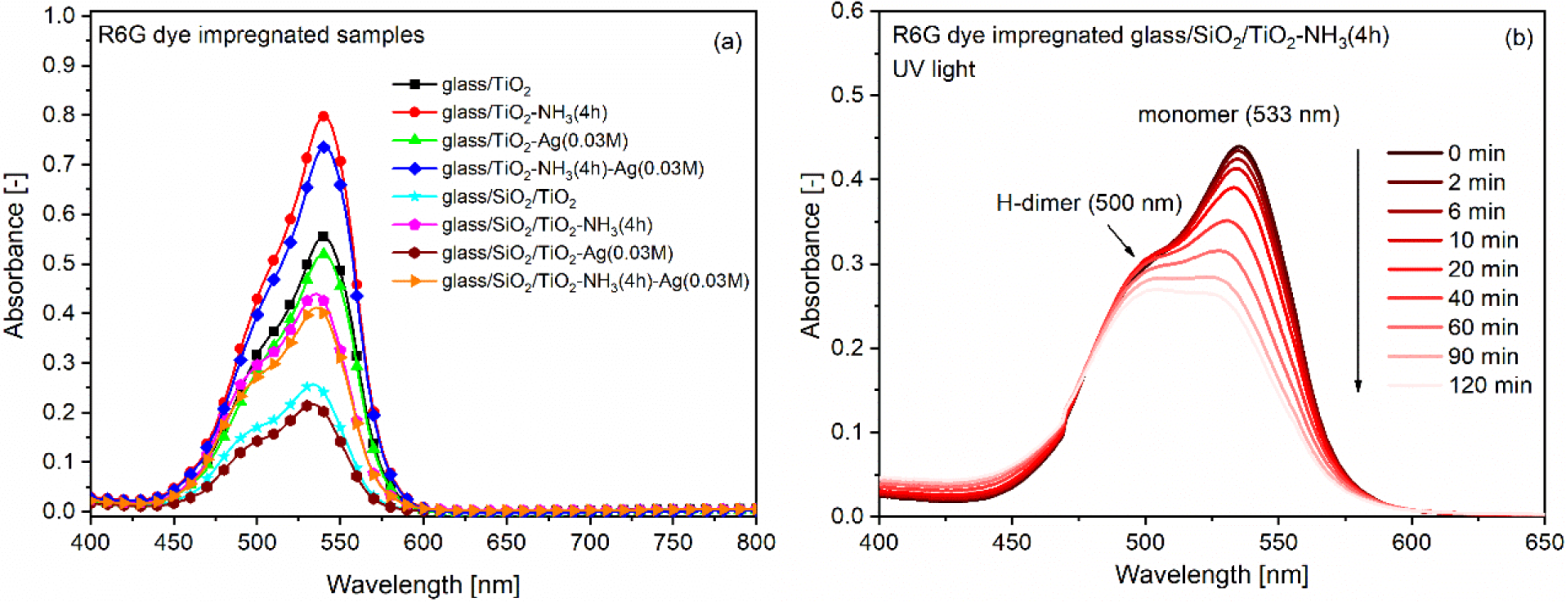
Absorbance spectra of the R6G dye-impregnated samples prior to irradiation (a) and the decrease in the absorbance of R6G dye molecules adsorbed in the pore system of the glass/SiO_2_/TiO_2_-NH_3_(4h) type sample under UV light irradiation (b).

In order to investigate the effect of aqueous ammonia vapor treatment on mesoporous TiO_2_ sol–gel coatings and their photocatalytic activity, the time-dependent decrease in the absorbance of R6G molecules impregnated in the pore system of the coatings was monitored under UV and visible light irradiation at the solid–air interface.^7^ Based on the measurements, the relative absorbance (*A*/*A*_0_) was determined as the ratio of the absorbance maximum (*A*) measured at a given time for the dye monomer peak (around 533 nm) to the peak maximum recorded at time zero (*A*_0_). Figure 6b shows the reduction in absorbance of R6G dye molecules, which had been preimpregnated into the pore system of the glass/SiO_2_/TiO_2_-NH_3_(4h) type sample, as a result of UV irradiation. In addition to the main absorption maximum at 533 nm, which corresponds to the monomer form of R6G, a shoulder at approximately 500 nm is also observed, attributed to the H-type dimer form of R6G (Figure 6b). It is clearly visible that the degradation of the monomer is faster than that of the dimer, in agreement with our previous findings.^7^

The relative absorbance decrease of the R6G dye monomer form in the pore system of the TiO_2_ coatings, induced by UV and visible light irradiation as a function of irradiation time, can be seen in Figure 7a,b, respectively. Figure 7c,d shows the relative surface area-normalized specific absorbance values as a function of irradiation time under UV (c) and visible light (d) irradiation. Whereas the relative absorbance–irradiation time representation reflects the overall dye decomposition behavior of the prepared samples, the relative surface area-normalized specific absorbance-irradiation time graphs provide a more accurate measure of the intrinsic activity of the photocatalysts.

**Figure 7:**
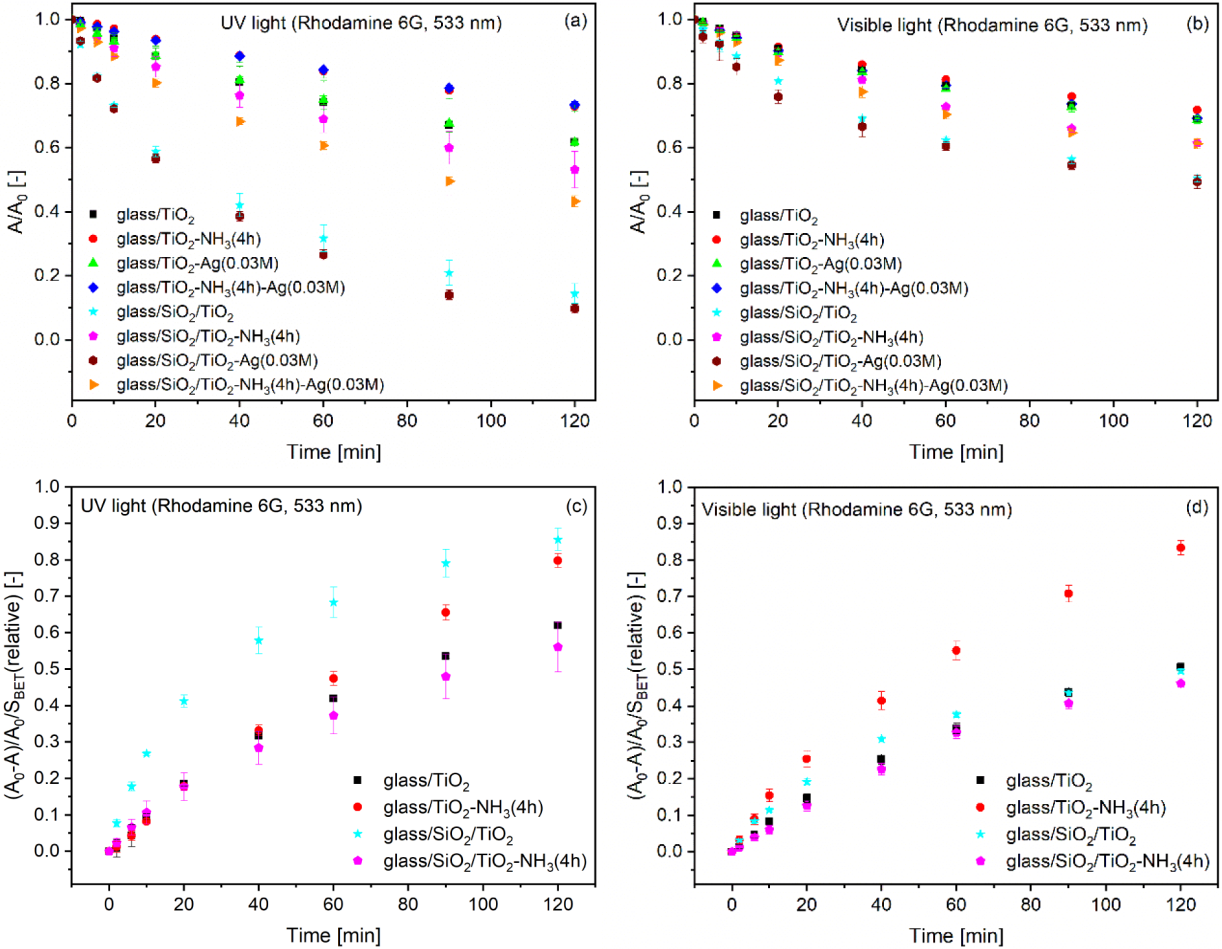
The change in the relative absorbance (a,b) and the relative surface area-normalized specific absorbance (c,d) values as a function of irradiation time under UV light (a,c) and visible light (b,d) for the investigated Rhodamine 6G dye-impregnated samples. Absorbance values were measured at 533 nm, and SBET values were determined by ellipsometric porosimetry.

The treatment of the samples in an ammonia vapor atmosphere reduced the photocatalytic activity under both UV and visible light irradiation (Figure 7a,b). This is presumably due to differences in the porosity of the coatings. Colloidal aging in an ammonia vapor atmosphere led to an increased pore volume, a reduced specific surface area (Table 6), and a higher proportion of dye molecules trapped within the pores (Figure 7a). The two-layered samples with a barrier SiO_2_ coating show higher photocatalytic activity compared to their monolayered counterparts under both UV and visible light irradiation (see Figure 7a,b). Based on the XPS analysis results, the barrier SiO_2_ coating present between the glass substrate and the TiO_2_ coatings significantly reduced the Na^+^ ion content within the TiO_2_ coatings (Table 1). Considering these results, the lower photoactivity of the monolayered TiO_2_ samples under UV irradiation can be attributed to the negative effect of Na^+^ ions (which have diffused from the glass substrate into the coatings during their heat treatment) on their photocatalytic activity.^7^ The effect of silver on photoactivity is more pronounced under UV light irradiation; however, compared to the effect of ammonia vapor treatment, it has a more moderate impact on the photocatalytic performance (Figure 7a,b).

It can be observed that TiO_2_ coatings, even without a silver dopant, exhibit significant photocatalytic activity in the visible range (Figure 7b). This appears to contradict the fact that undoped TiO_2_ is not photoactive in this range.^49,50^ However, the R6G dye can extend the photocatalytic activity of TiO_2_ into the visible light range through its sensitizing effect,^7^ which likely also prevails in the present study.

According to the graphs presenting the photocatalytic behavior of the prepared samples under UV light irradiation (Figure 7a,c), the as-prepared two-layer (glass/SiO_2_/TiO_2_) sample (with minimal Na of Na^+^ ions in the TiO_2_ layer) showed the highest performance. However, the aqueous ammonia vapor treatment reduced its activity (Figure 7a,c). It is interesting to note that the ammonia treatment enhanced the activity of the monolayer photocatalyst (Figure 7c). By the end of the investigated time range, the photocatalytic rate of the ammonia-treated monolayer sample (glass/TiO_2_-NH_3_(4h)) reached that of the two-layer sample (glass/SiO_2_/TiO_2_) with the highest photocatalytic activity (Figure 7c). Conversely, under visible light irradiation, the ammonia-treated monolayer catalyst performed the best, despite the Na^+^ ion content in the TiO_2_ layer (Figure 7d). The results indicate that this dye-sensitized pathway of photodegradation is either insensitive to or only marginally affected by the Na^+^ ion content of the coatings. A comprehensive understanding of the photocatalytic performance exhibited by the ammonia-treated mono- and two-layer coatings requires further systematic investigation.

## Conclusions

The structural rearrangement of mesoporous TiO_2_ sol–gel coatings via aqueous ammonia vapor-induced pseudomorphic transformation is reported in this work. The coatings, which still contained the molecular template (Pluronic P123), were aged in an aqueous ammonia vapor atmosphere immediately after deposition onto various substrates (glass, silica-coated glass, and silica-coated silicon), resulting in significant pore structure rearrangement in all cases.

The structural changes were investigated and characterized using thin-layer optical methods (UV–vis spectroscopy and ellipsometric porosimetry) and transmission electron microscopy. Most importantly, from the perspective of improving light transmittance, the aqueous ammonia vapor treatment proved to be more effective than the aqueous vapor treatments previously reported in the literature: just a 4-h exposure to ammonia vapor resulted in a 1.25% higher average transmittance increase compared to a 4-day treatment in an aqueous vapor atmosphere on a silica-coated glass substrate. The maximum light transmittance increase, compared to that of the bare glass substrate, was found to be 3.25% as a result of the pseudomorphic transformation in the investigated systems. According to this, compared to the untreated samples, the coatings exhibited a notable increase in porosity (decrease in the effective refractive index), accompanied by a reduction in specific surface area on all substrates. Interestingly, while ammonia treatment reduced the intrinsic photoactivity of the two-layer photocatalyst, the same treatment improved the activity of the monolayer one.

## Supplementary Material



## Notes

XPS spectra; additional UV–vis transmittance curves of the samples, and their optical properties determined by analyzing the transmittance curves (PDF)

## References

[1] Yao, K. J. A. ; Hartiti, B. ; Konan, F. K. ; Ziti, A. ; Batan, A. ; Labrim, H. ; Laazizi, A. ; Aka, B. ; Thevenin, P. Sol-Gel Deposition of TiO2 Thin Films by Spin Coating for Photovoltaic Applications: Effect Of Acetylacetone Stabilizer On Structural And Optical Properties. In Materials Today: Proceedings, Elsevier, 2024; p. S221478532400066X. DOI: 10.1016/j.matpr.2024.02.003.

[2] Bai Y., Mora-Seró I., De Angelis F., Bisquert J., Wang P. (2014). Titanium Dioxide
Nanomaterials for Photovoltaic Applications. Chem. Rev..

[3] Grätzel M. (2001). Sol-Gel Processed
TiO_2_ Films for Photovoltaic Applications. J. sol-Gel Sci. Technol..

[4] Lukong V. T., Mouchou R. T., Enebe G. C., Ukoba K., Jen T. C. (2022). Deposition
and Characterization of Self-Cleaning TiO_2_ Thin Films for
Photovoltaic Application. Mater. Today Proc..

[5] Agarwala S., Kevin M., Wong A. S. W., Peh C. K. N., Thavasi V., Ho G. W. (2010). Mesophase Ordering
of TiO_2_ Film with High Surface Area
and Strong Light Harvesting for Dye-Sensitized Solar Cell. ACS Appl. Mater. Interfaces.

[6] Gayle A. J., Lenef J. D., Huff P. A., Wang J., Fu F., Dadheech G., Dasgupta N. P. (2022). Visible-Light-Driven
Photocatalysts
for Self-Cleaning Transparent Surfaces. Langmuir.

[7] Tegze B., Albert E., Fodor B., Sáfrán G., Hórvölgyi Z. (2019). Photoinduced Processes of Adsorbed
and Associated Dye Molecules in Mesoporous Titania Coatings. Dyes Pigm..

[8] Obregón S., Rodríguez-González V. (2022). Photocatalytic
TiO_2_ Thin
Films and Coatings Prepared by Sol–Gel Processing: A Brief
Review. J. Sol-Gel Sci. Technol..

[9] Tegze B., Albert E., Dikó B., Nagy N., Rácz A., Sáfrán G., Sulyok A., Hórvölgyi Z. (2021). Effect of
Silver Modification on the Photoactivity of Titania Coatings with
Different Pore Structures. Nanomaterials.

[10] Kéri O., Kócs L., Hórvölgyi Z., Baji Z., László K., Takáts V., Erdélyi Z., Szilágyi I. M. (2019). Photocatalytically
Active Amorphous and Crystalline
TiO_2_ Prepared by Atomic Layer Deposition. Period. Polytech., Chem. Eng..

[11] Fónagy O., Hegedűs P., Szabó-Bárdos E., Dobrádi A., Horváth O. (2017). Improvement Possibilities of Heterogeneous Photocatalysis
with the Aim of In-Field Use. Hung. J. Ind.
Chem..

[12] Mogyorósi K., Farkas A., Dékány I., Ilisz I., Dombi A. (2002). TiO_2_ -Based Photocatalytic Degradation of 2-Chlorophenol
Adsorbed on Hydrophobic Clay. Environ. Sci.
Technol..

[13] Doubi Y., Hartiti B., Labrim H., Fadili S., Tahri M., Belafhaili A., Siadat M., Thevenin P. (2021). Experimental Study
of Properties of TiO_2_ Thin Films Deposited by Spray Pyrolysis
for Future Sensory Applications. Appl. Phys.
A: Mater. Sci. Process..

[14] Danish M. S. S., Estrella L. L., Alemaida I. M. A., Lisin A., Moiseev N., Ahmadi M., Nazari M., Wali M., Zaheb H., Senjyu T. (2021). Photocatalytic Applications of Metal
Oxides for Sustainable
Environmental Remediation. Metals.

[15] Akhavan O., Ghaderi E. (2010). Self-Accumulated Ag
Nanoparticles on Mesoporous TiO2
Thin Film with High Bactericidal Activities. Surf. Coat. Technol..

[16] Albert E., Albouy P. A., Ayral A., Basa P., Csík G., Nagy N., Roualdès S., Rouessac V., Sáfrán G., Suhajda Á., Zolnai Z., Hórvölgyi Z. (2015). Antibacterial
Properties of Ag–TiO_2_ Composite Sol–Gel Coatings. RSC Adv..

[17] Fónagy O., Kovács M., Szabó-Bárdos E., Csicsor-Kulcsár P., Fodor L., Horváth O. (2025). Mechanochemically Modified TiO_2_ Photocatalysts: Combination of Visible-Light Excitability
and Antibacterial Effect. Catalysts.

[18] Aziz W. J., Ramizy A., Ibrahim K., Hassan Z., Omar K. (2011). The Effect
of Anti-Reflection Coating of Porous Silicon on Solar Cells Efficiency. Optik.

[19] Jilavi M. H., Mousavi S. H., Müller T. S., De Oliveira P. W. (2018). Dual Functional
Porous Anti-Reflective Coatings with a Photocatalytic Effect Based
on a Single Layer System. Appl. Surf. Sci..

[20] Yao L., He J. (2014). Recent Progress in
Antireflection and Self-Cleaning Technology –
From Surface Engineering to Functional Surfaces. Prog. Mater. Sci..

[21] Rad A. S., Afshar A., Azadeh M. (2020). Anti-Reflection and
Self-Cleaning
Meso-Porous TiO_2_ Coatings as Solar Systems Protective Layer:
Investigation of Effect of Porosity and Roughness. Opt. Mater..

[22] Reber M. J., Brühwiler D. (2015). Mesoporous Hybrid Materials by Simultaneous Pseudomorphic
Transformation and Functionalization of Silica Microspheres. Part. Part. Syst. Charact..

[23] Boudot M., Gaud V., Louarn M., Selmane M., Grosso D. (2014). Sol–Gel
Based Hydrophobic Antireflective Coatings on Organic Substrates: A
Detailed Investigation of Ammonia Vapor Treatment (AVT). Chem. Mater..

[24] Kócs L., Tegze B., Albert E., Major C., Szalai A., Fodor B., Basa P., Sáfrán G., Hórvölgyi Z. (2021). Ammonia-Vapour-Induced
Two-Layer
Transformation of Mesoporous Silica Coatings on Various Substrates. Vacuum.

[25] Das S., Roy S., Patra A., Biswas P. K. (2003). Study of Refractive Index and Physical
Thickness of Porous Silica Films with Ageing in Hydrated Ammonia and
Air. Mater. Lett..

[26] Sakatani Y., Grosso D., Nicole L., Boissière C., de Aa soler-Illia G.
J., Sanchez C. (2006). Optimised
Photocatalytic Activity
of Grid-like Mesoporous TiO_2_ Films: Effect of Crystallinity,
Pore Size Distribution, and Pore Accessibility. J. Mater. Chem..

[27] Grosso D., Soler-Illia G. J. D. A., Crepaldi E. L., Cagnol F., Sinturel C., Bourgeois A., Brunet-Bruneau A., Amenitsch H., Albouy P. A., Sanchez C. (2003). Highly Porous
TiO_2_ Anatase
Optical Thin Films with Cubic Mesostructure Stabilized at 700 °C. Chem. Mater..

[28] Soler-Illia G. J. A. A., Angelomé P. C., Fuertes M. C., Grosso D., Boissiere C. (2012). Critical Aspects in the Production of Periodically
Ordered Mesoporous Titania Thin Films. Nanoscale.

[29] Crepaldi E. L., Soler-Illia G. J. D. A. A., Grosso D., Cagnol F., Ribot F., Sanchez C. (2003). Controlled
Formation of Highly Organized
Mesoporous Titania Thin Films: From Mesostructured Hybrids to Mesoporous
Nanoanatase TiO_2_. J. Am. Chem. Soc..

[30] Cedillo-González E. I., Mugoni C., Montorsi M., Siligardi C. (2016). Evaluation
of the Correlations between Temperature, Humidity, Incident UV Light
and the Photocatalytic Activity of TiO_2_ Films Using a Rationale
Approach. Appl. Surf. Sci..

[31] Wang P., Chen D., Tang F.-Q. (2006). Preparation
of Titania-Coated Polystyrene
Particles in Mixed Solvents by Ammonia Catalysis. Langmuir.

[32] Verma J., Geng Y., Yan Y., Goel S. (2025). Fabrication of Titania
Nanoparticles with Reduced Energy Footprint Using a Novel Low-Temperature
Peptization Method. Prog. Org. Coat..

[33] Yun H., Miyazawa K., Honma I., Zhou H., Kuwabara M. (2003). Synthesis
of Semicrystallized Mesoporous TiO_2_ Thin Films Using Triblock
Copolymer Templates. Mater. Sci. Eng..

[34] Klotz M., Ayral A., Guizard C., Cot L. (2000). Synthesis Conditions
for Hexagonal Mesoporous Silica Layers. J. Mater.
Chem..

[35] Novotna P., Krysa J., Maixner J., Kluson P., Novak P. (2010). Photocatalytic
Activity of Sol–Gel TiO2 Thin Films Deposited on Soda Lime
Glass and Soda Lime Glass Precoated with a SiO_2_ Layer. Surf. Coat. Technol..

[36] Kócs L., Albert E., Tegze B., Kabai-Faix M., Major C., Szalai A., Basa P., Hórvölgyi Z. (2017). Silica Sol-Gel
Coatings with Improved Light Transmittance and Stability. Period. Polytech., Chem. Eng..

[37] Aureau D., Ridier K., Bérini B., Dumont Y., Keller N., Vigneron J., Bouttemy M., Etcheberry A., Fouchet A. (2016). Advanced Analysis Tool for X-Ray
Photoelectron Spectroscopy
Profiling: Cleaning of Perovskite SrTiO_3_ Oxide Surface
Using Argon Cluster Ion Source. Thin Solid Films.

[38] Hild E., Deák A., Naszályi L., Sepsi Ö., Ábrahám N., Hórvölgyi Z. (2007). Use of the Optical Admittance Function
and Its WKB Approximation to Simulate and Evaluate Transmittance Spectra
of Graded-Index Colloidal Films. J. Opt. A:
Pure Appl. Opt..

[39] Albert E., Basa P., Fodor B., Keresztes Z., Madarász J., Márton P., Olasz D., Rácz A. S., Sáfrán G., Szabó T., Tegze B., Höltzl T., Hórvölgyi Z. (2025). Experimental and Computational Synthesis
of TiO_2_ Sol–Gel Coatings. Langmuir.

[40] McClellan A. L., Harnsberger H. F. (1967). Cross-Sectional Areas of Molecules
Adsorbed on Solid
Surfaces. J. Colloid Interface Sci..

[41] Ghazzal M. N., Kebaili H., Joseph M., Debecker D. P., Eloy P., De Coninck J., Gaigneaux E. M. (2012). Photocatalytic Degradation of Rhodamine
6G on Mesoporous Titania Films: Combined Effect of Texture and Dye
Aggregation Forms. Appl. Catal., B.

[42] Vogel R., Meredith P., Harvey M. D., Rubinsztein-Dunlop H. (2004). Absorption
and Fluorescence Spectroscopy of Rhodamine 6G in Titanium Dioxide
Nanocomposites. Spectrochim. Acta, Part A.

[43] Briggs, D. Handbook of X-ray Photoelectron Spectroscopy C. D. Wanger, W. M. Riggs, L. E. Davis, J. F. Moulder and G. E.Muilenberg Perkin-Elmer Corp., Physical Electronics Division, Eden Prairie, Minnesota, USA, 1979. 190 Pp. $195. Surf. Interface Anal., 1981, 3, 4, 10.1002/sia.740030412.

[44] Lützenkirchen-Hecht D. (2011). Electrochemically
Grown Silver Oxide (Ag_2_O) by XPS. Surf. Sci. Spectra.

[45] Lützenkirchen-Hecht D. (2011). Anodic Silver
Oxide (AgO) Layers by XPS. Surf. Sci. Spectra.

[46] Liu R., Wang P., Wang X., Yu H., Yu J. (2012). UV- and Visible-Light
Photocatalytic Activity of Simultaneously Deposited and Doped Ag/Ag­(I)-TiO_2_ Photocatalyst. J. Phys. Chem. C.

[47] Szabó-Bárdos E., Czili H., Horváth A. (2003). Photocatalytic Oxidation of Oxalic
Acid Enhanced by Silver Deposition on a TiO_2_ Surface. J. Photochem. Photobiol., A.

[48] Akhavan O. (2009). Lasting Antibacterial
Activities of Ag–TiO_2_/Ag/a-TiO_2_ Nanocomposite
Thin Film Photocatalysts under Solar Light Irradiation. J. Colloid Interface Sci..

[49] Chakraborty A. K., Ganguli S., Sabur M. A. (2023). Nitrogen
Doped Titanium Dioxide (N-TiO2):
Electronic Band Structure, Visible Light Harvesting and Photocatalytic
Applications. J. Water Process. Eng..

[50] Cuadra J. G., Molina-Prados S., Mínguez-Vega G., Estrada A., Trindade T., Oliveira C., Seabra M. P., Labrincha J., Porcar S., Cadena R. (2023). Multifunctional Silver-Coated
Transparent TiO_2_ Thin Films for Photocatalytic and Antimicrobial
Applications. Appl. Surf. Sci..

