# Correction to “Ammonia Vapor-Induced Pseudomorphic Transformation of Mesoporous TiO_2_ Sol-Gel Coatings”

**DOI:** 10.1021/acsomega.6c06859

**Published:** 2026-07-01

**Authors:** Adrienn Márta Bors, János Madarász, Norbert Nagy, Adél Sarolta Rácz, György Sáfrán, Dániel Olasz, Zoltán Hórvölgyi, Emőke Albert

Figure 1 in the published article was presented incorrectly. The corrected Figure 1 is provided below. The figure caption remains unchanged and is reproduced below for completeness.

**Figure 1:**
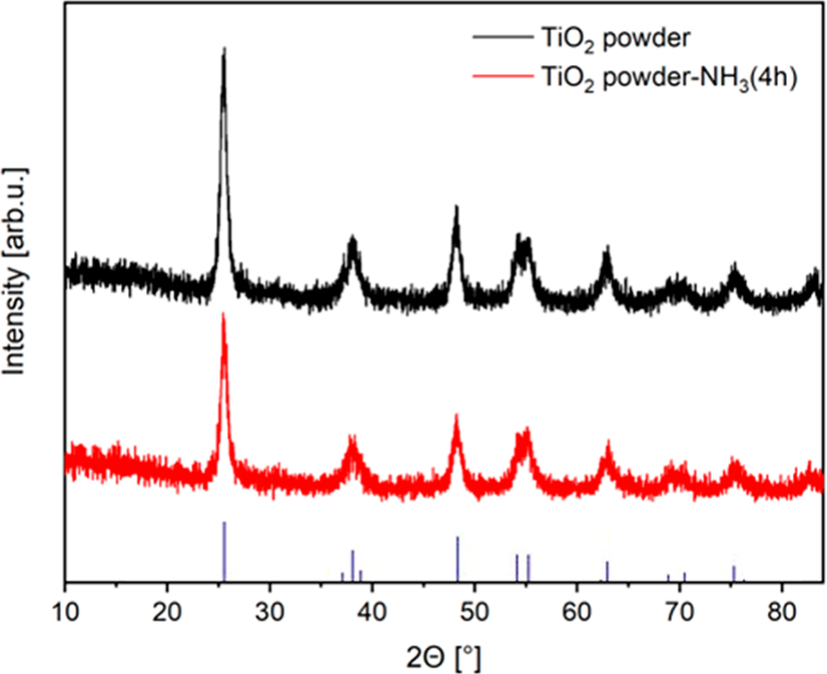
XRD patterns of the untreated TiO_2_ powder sample (black pattern) and the TiO_2_ powder sample treated under a 2 M aqueous NH_3_ solution atmosphere for 4 h (red pattern) are shown. The positions of the standard reflections for anatase [PDF 98-000-9852] are also shown as vertical bars at the bottom. The diagram is shifted along the intensity axis for better visibility.

This correction is limited to the graphical representation of Figure 1 and does not affect the underlying experimental results, the discussion, or the conclusions of the manuscript.

