# Supplementary material for: Ammonia Vapor-Induced Pseudomorphic Transformation of Mesoporous TiO2 Sol–Gel Coatings
Source: ACS Omega. 2025 Jul 28;10(31):35029–42. doi: 10.1021/acsomega.5c04483 (PMC12355428; doi:10.1021/acsomega.5c04483)
Supplement: Supplementary file 1 [file ao5c04483_si_001.pdf]

SUPPORTING INFORMATION

Ammonia Vapour-Induced Pseudomorphic Transformation of Mesoporous TiO_2 Sol-Gel Coatings

Adrienn Márta Bors[1]*, János Madarász*[2]*, Norbert Nagy*[3]*, Adél Sarolta Rácz*[3]*, György Sáfrán*[3]*, Dániel Olasz*[3]*, Zoltán Hórvölgyi*[1,*]*, Emőke Albert*[1,*]

[1]Department of Physical Chemistry and Materials Science, Budapest University of Technology and Economics, Műegyetem rkp. 3, 1111 Budapest, Hungary

[*]E-mail: horvolgyi.zoltan@vbk.bme.hu (Zoltán Hórvölgyi)

[*]E-mail: albert.emoke@vbk.bme.hu (Emőke Albert)

[2]Department of Inorganic and Analytical Chemistry, Budapest University of Technology and Economics, Műegyetem rkp. 3, 1111 Budapest, Hungary

[3]HUN-REN Centre for Energy Research, Institute of Technical Physics and Materials Science, Konkoly-Thege Miklós út 29-33, 1121 Budapest, Hungary

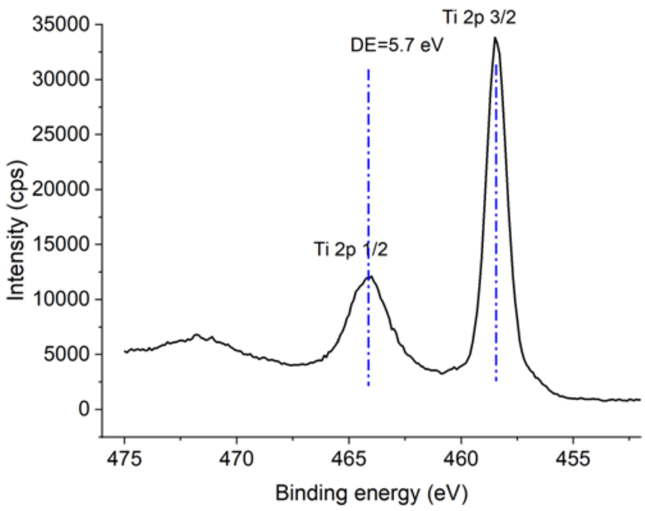

Figure SI1: Ti 2p peak for the glass/SiO_2/TiO_2-NH_3(4h)-Ag(0.03M) type sample after cluster sputtering.

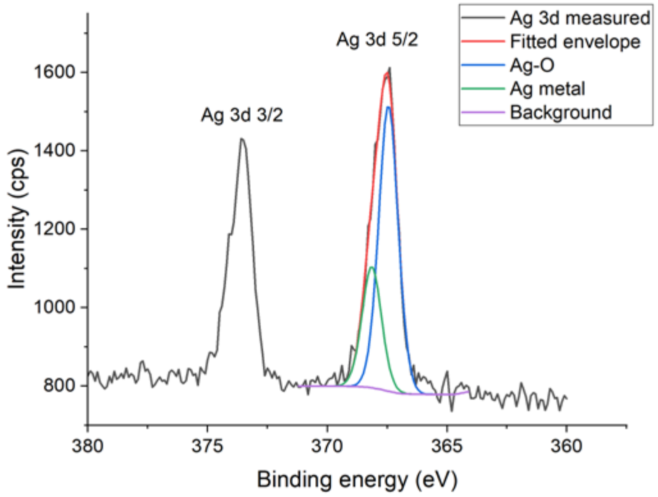

Figure SI2: Decomposed $Ag3d_{5/2}$ peak after cluster sputtering for the glass/SiO_2/TiO_2-NH_3(4h)-Ag(0.03M) type sample.

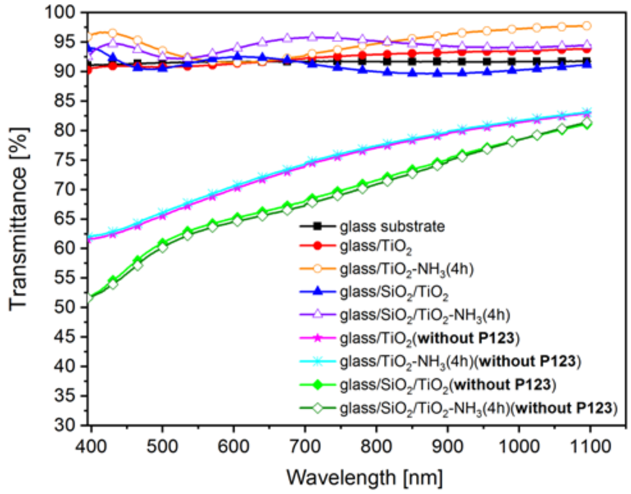

Figure SI3: Representative transmittance spectra of the bare glass substrate, the monolayered (glass/TiO_2, glass/TiO_2-NH_3(4h), glass/TiO_2(without P123), glass/TiO_2-NH_3(4h)(without P123)), and the two-layered (glass/SiO_2/TiO_2, glass/SiO_2/TiO_2-NH_3(4h), glass/SiO_2/TiO_2(without P123), glass/ SiO_2/TiO_2-NH_3(4h)(without P123)) samples, untreated (glass/TiO_2, glass/TiO_2(without P123), glass/SiO_2/TiO_2, glass/SiO_2/TiO_2(without P123)) or treated (glass/TiO_2-NH_3(4h), glass/TiO_2-NH_3(4h)(without P123), glass/SiO_2/TiO_2-NH_3(4h), glass/SiO_2/TiO_2-NH_3(4h)(without P123) in aqueous ammonia vapour atmosphere for 4 hours, deposited onto glass substrates from TiO_2 precursor suspension without (glass/TiO_2(without P123), glass/SiO_2/TiO_2(without P123), glass/TiO_2-NH_3(4h)(without P123), glass/SiO_2/TiO_2-NH_3(4h)(without P123)) and with (glass/TiO_2, glass/SiO_2/TiO_2, glass/TiO_2-NH_3(4h), glass/SiO_2/TiO_2-NH_3(4h)) Pluronic P123 content.

Table SI1: Effective refractive index (n), thickness (d), calculated porosity (P, Lorentz-Lorenz) values of the TiO_2 coatings of the investigated samples, deposited from TiO_2 precursor sols without or with Pluronic P123 content, determined from their UV-Vis transmittance spectra. The table also contains the average transmittance increment (ATI) values of the samples, determined for the 400-800 nm wavelength range, compared to their bare glass substrates.

**Sample**<br>**(prepared from precursor sol without/with Pluronic P123)**	**n [-] (632.8 nm)**<br>**TiO_2**	**d [nm]**<br>**TiO_2**	**P [%]**<br>**TiO_2**	**ATI [%]**<br>**(400-800 nm)**
glass/TiO_2<br>(without Pluronic P123)	2.0031 ± 0.0063	52 ± 1	18 ± 0	-21.80 ± 0.38
glass/TiO_2<br>(with Pluronic P123)	1.5137 ± 0.0039	140 ± 2	51 ± 0	0.03 ± 0.01
glass/TiO_2-NH_3(4h)<br>(without Pluronic P123)	2.0001 ± 0.0051	52 ± 2	18 ± 0	-21.79 ± 0.11
glass/TiO_2-NH_3(4h)<br>(with Pluronic P123)	1.3685 ± 0.0035	221 ± 2	63 ± 0	1.82 ± 0.08
glass/SiO_2/TiO_2<br>(without Pluronic P123)	2.1422 ± 0.0098	43 ± 2	11 ± 0	-26.89 ± 0.65
glass/SiO_2/TiO_2<br>(with Pluronic P123)	1.5266 ± 0.0277	131 ± 10	50 ± 2	-0.57 ± 1.30
glass/SiO_2/TiO_2-NH_3(4h)<br>(without Pluronic P123)	2.1137 ± 0.0282	46 ± 2	13 ± 1	-27.76 ± 0.50
glass/SiO_2/TiO_2-NH_3(4h)<br>(with Pluronic P123)	1.3998 ± 0.0090	194 ± 2	61 ± 1	2.93 ± 0.17
